# Extending gene ontology in the context of extracellular RNA and vesicle communication

**DOI:** 10.1186/s13326-016-0061-5

**Published:** 2016-04-12

**Authors:** Kei-Hoi Cheung, Shivakumar Keerthikumar, Paola Roncaglia, Sai Lakshmi Subramanian, Matthew E. Roth, Monisha Samuel, Sushma Anand, Lahiru Gangoda, Stephen Gould, Roger Alexander, David Galas, Mark B. Gerstein, Andrew F. Hill, Robert R. Kitchen, Jan Lötvall, Tushar Patel, Dena C. Procaccini, Peter Quesenberry, Joel Rozowsky, Robert L. Raffai, Aleksandra Shypitsyna, Andrew I. Su, Clotilde Théry, Kasey Vickers, Marca H.M. Wauben, Suresh Mathivanan, Aleksandar Milosavljevic, Louise C. Laurent

**Affiliations:** Department of Emergency Medicine, Yale Center for Medical Informatics, Yale University School of Medicine, New Haven, CT USA; VA Connecticut Healthcare System, West Haven, CT USA; Department of Biochemistry and Genetics, La Trobe Institute for Molecular Science, La Trobe University, Melbourne, VIC 3086 Australia; European Bioinformatics Institute (EMBL-EBI), European Molecular Biology Laboratory, Wellcome Trust Genome Campus, Hinxton, Cambridge, CB10 1SD UK; Bioinformatics Research Laboratory, Department of Molecular & Human Genetics, Baylor College of Medicine, Houston, TX USA; Department of Biological Chemistry, Johns Hopkins University School of Medicine, Baltimore, MD USA; Pacific Northwest Diabetes Research Institute, Seattle, WA USA; Department of Molecular Biophysics and Biochemistry, Yale University, New Haven, CT USA; Department of Computer Science, Yale University, New Haven, CT USA; Program in Computational Biology and Bioinformatics, Yale University, New Haven, CT USA; University of Gothenburg, Gothenburg, Sweden; Mayo Clinic, Jacksonville, FL USA; Division of Neuroscience and Behavior, National Institute on Drug Abuse (NIDA), Rockville, MD USA; University Medicine Comprehensive Cancer Center, Providence, RI USA; Department of Surgery, University of California San Francisco and VA Medical Center, San Francisco, CA USA; Department of Molecular and Experimental Medicine, The Scripps Research Institute, La Jolla, CA USA; Institut Curie, PSL Research University, INSERM U932, Paris, France; Department of Medicine, Vanderbilt University School of Medicine, Nashville, TN USA; Department of Biochemistry & Cell Biology, Utrecht University, Utrecht, Netherlands; Department of Reproductive Medicine, University of California, San Diego, La Jolla, CA USA; Extracellular RNA Communication Consortium (ERCC), ᅟ, ᅟ; Gene Ontology Consortium (GOC), ᅟ, ᅟ; American Society for Exosomes and Microvesicles (ASEMV), ᅟ, ᅟ; International Society for Extracellular Vesicles (ISEV), ᅟ, ᅟ

**Keywords:** Ontology, Extracellular RNA, Extracellular vesicle, Metadata, Faceted search, Atlas

## Abstract

**Background:**

To address the lack of standard terminology to describe extracellular RNA (exRNA) data/metadata, we have launched an inter-community effort to extend the Gene Ontology (GO) with subcellular structure concepts relevant to the exRNA domain. By extending GO in this manner, the exRNA data/metadata will be more easily annotated and queried because it will be based on a shared set of terms and relationships relevant to extracellular research.

**Methods:**

By following a consensus-building process, we have worked with several academic societies/consortia, including ERCC, ISEV, and ASEMV, to identify and approve a set of exRNA and extracellular vesicle-related terms and relationships that have been incorporated into GO. In addition, we have initiated an ongoing process of extractions of gene product annotations associated with these terms from Vesiclepedia and ExoCarta, conversion of the extracted annotations to Gene Association File (GAF) format for batch submission to GO, and curation of the submitted annotations by the GO Consortium. As a use case, we have incorporated some of the GO terms into annotations of samples from the exRNA Atlas and implemented a faceted search interface based on such annotations.

**Results:**

We have added 7 new terms and modified 9 existing terms (along with their synonyms and relationships) to GO. Additionally, 18,695 unique coding gene products (mRNAs and proteins) and 963 unique non-coding gene products (ncRNAs) which are associated with the terms: “extracellular vesicle”, “extracellular exosome”, “apoptotic body”, and “microvesicle” were extracted from ExoCarta and Vesiclepedia. These annotations are currently being processed for submission to GO.

**Conclusions:**

As an inter-community effort, we have made a substantial update to GO in the exRNA context. We have also demonstrated the utility of some of the new GO terms for sample annotation and metadata search.

## Background

Extracellular RNAs (exRNAs) are broadly defined as RNAs that are present in the acellular portions of biofluids, such as body fluids (blood, cerebrospinal fluid, bile, lymph, vitreous humour, amniotic fluid, ascites, pleural, pericardial and peritoneal fluids, etc.), secretions (saliva, urine, sweat, tears, milk, seminal fluid, etc.), and cell and tissue culture supernatants. RNA sequencing analyses of exRNAs demonstrate that they represent almost the entire range of cellular RNA species, including rRNAs, tRNAs, mRNAs, miRNAs, piRNAs, lncRNAs, and circular RNAs ([1–5]). However, the profiles of cellular and exRNAs are not identical, as some cellular RNAs appear to be highly enriched in the exRNA fraction, while others appear to be significantly underrepresented, and still others lie between these extremes of enrichment or exclusion.

Among the many reasons cells might release exRNAs, perhaps the most intriguing possibility is that exRNAs might contribute to intercellular communication. Export of exRNAs may also be used to eliminate undesired RNAs from the originating cell. Finally, some exRNAs might be generated in a nonspecific manner, either by living cells (e.g. by a ‘bulk flow’ process) or as a consequence of cell death (reviewed in [6]).

ExRNAs appear to be universally associated with carrier vehicles, likely due to the rapid degradation of unprotected RNAs in biofluids ([4, 7–10]). The biochemical properties of these carriers are likely to be the primary determinant of the types and specific identities of exRNAs that are secreted from cells, as well as their stability in the extracellular milieu. The first exRNA carriers identified were RNA viruses, which carry not only viral RNAs, but also varying levels of host-encoded RNAs. For example, retroviruses typically carry two host tRNAs and sub-stoichiometric levels of host mRNAs from the cell in addition to two copies of the viral RNA genome and key viral proteins ([11–16]). In fact, retrovirally infected cells produce more “empty” virus-like particles (VLPs) than infectious virions. These VLPs have failed to encapsulate the viral RNA genome, but can carry as much as 10 kb of host-encoded RNA ([17]). More recently, it has been discovered that virally uninfected cells also release RNAs into the extracellular space, and that these exRNAs are associated with extracellular vesicles (EVs), lipoproteins (LPPs, most commonly HDLs ([10, 18]), LDLs ([18, 19])), and ribonucleoprotein particles (RNPs, most commonly Ago2-containing RNPs ([9, 20]). While the biogenic mechanisms underlying the release of exRNA-containing EVs, LPPs, and RNPs are still being investigated, it is clear that they are not generated by a mechanism that is common to all of them.

Evidence for EVs and exRNA was first provided more than seventy-five years ago by Albert Claude’s observation that uninfected chick and mammalian cells release RNA-containing vesicles ([21]). However, these non-viral EVs remained largely uninvestigated for decades. EVs re-entered the literature in the late 1960’s with descriptions of calcifying matrix vesicles released by chondrocytes ([22]) and vesicular ‘dust’ released by platelets ([23]). These and other such vesicles are now commonly referred to as ‘exosomes’, a term coined by Trams et al. in 1981 ([24]) to refer to secreted vesicles that “*may serve a physiologic function*”, including both small vesicles of ~100 nm in diameter, and larger vesicles of ~600 nm diameter or greater.

This first definition of the term ‘exosome’ has been subsequently overlooked at least twice, first in 1987 by investigators studying the vesicular secretion of the transferrin receptor, who adopted a more restrictive definition of the term, conflating it with a delayed mode of vesicle secretion in which the vesicles bud at endosome membranes to create a multivesicular body (MVB), followed later by MVB fusion with the plasma membrane to release the vesicles into the extracellular space ([25]). In 1997, investigators studying an RNA-degrading protein complex adopted the term ‘exosome’, this time for an entirely unrelated intracellular biochemical entity ([26]). Not surprisingly, other investigators have come to different conclusions about which definition holds scientific precedent, resulting in variable use of the term ‘exosome’ in different laboratories. EV-related nomenclatures are further complicated by the common use of additional terms for secreted vesicles that are variably associated with different biophysical properties or biogenesis pathways, as wells as terms based on the cell type or tissue of origin. The former include ‘ectosomes’ (which refer to EVs that are produced by budding from the plasma membrane ([27, 28]) and ‘microvesicles’ (which are frequently operationally defined as EVs that pellet at moderate centrifugation speeds (~10,000–20,000 xg) [29]), while the latter include ‘prostasomes’ ([30]), ‘epididymosomes’ ([31]), ‘immunosomes’ ([32, 33]), ‘oncosomes’ ([34]), and ‘platelet dust’ ([23]). There are even some vesicle names that refer to observed biological activities (e.g. ‘tolerosomes’ ([35]) and ‘calcifying matrix vesicles’ ([22]).

The International Society for Extracellular Vesicles (ISEV) has previously attempted to clarify the nomenclature in this field. Its primary achievements have been to (1) introduce the term ‘extracellular vesicle (EV)’ as a general term intended to encompass all secreted vesicles, and encourage its broad acceptance, and (2) encourage the use of broad-definition terms until a more comprehensive understanding of the biogenesis and molecular compositions of different types of vesicles is developed. Given the inconsistent vesicle nomenclatures prior to these ISEV efforts, these were major advances. However, some inconsistencies still persist. For example, some investigators use the term ‘microvesicle’ for larger EVs (>200 nm diameter) and ‘exosome’ for smaller EVs (~30–200 nm diameter), while other investigators reject these size-based definitions and adopt a set of biogenic definitions in which ‘microvesicle’ describes vesicles that bud from the plasma membrane while ‘exosome’ describes vesicles that bud into endosomes and are secreted only later upon MVB fusion with the plasma membrane.

Therefore, investigators are currently forced to make a choice between these competing definitions, the first being practical but mechanistically barren, while the latter being impractical but mechanistically appealing. Unfortunately, there is as yet no unambiguous way to distinguish between vesicles that bud from the plasma membrane versus those that bud at the endosome membrane based on either biophysical properties or molecular content. This lack of standard nomenclatures creates i) a problem for individual researchers to annotate/share their data in an unambiguous manner and ii) a barrier to productive interactions with the broader research community, most prominently the biomedical, genomics, and computational biology communities. To address such issues, we initiated our metadata standard efforts within the Metadata Working Group (MWG) of the Extracellular RNA Communication Consortium (ERCC) funded by the National Institutes of Health (NIH). As part of these efforts, MWG matched metadata terms to existing biomedical ontologies and identified the exRNA-relevant terms that were absent from major ontologies such as the Gene Ontology (GO).

### Use of ontologies in metadata annotation

As described in [36], the MWG of the ERCC has developed the data and metadata standards for annotating exRNA profiling data for submission to the Data Management and Resource Repository (DMRR) of the ERCC. Particularly, a process has been established to submit experiment data to DMRR along with metadata in standard machine-readable formats (using Linked Data technologies). The standards cover metadata about donors, biosamples, experiments, studies, and analysis steps. Such metadata enable targeted selection of samples of interest (e.g. specific health condition of the donor, biofluid or cell/tissue type, library preparation method, and sequencing assay) for integrative analyses. The metadata also helps organize the data for efficient interactive as well as programmatic access (e.g. REST Application Programming Interfaces (APIs)).

The MWG identified existing biomedical ontologies accessible through the NCBO BioPortal [37] as a source of commonly accepted terms for annotating exRNA datasets. Such ontology-based metadata annotation does not only allow semantic retrieval/query of data in ERCC data sources, it also allows DMRR data to be integrated with other data sources with metadata annotated using the same ontologies. For ontologies to be useful for biological applications, it is critical that the relevant ontologies contain meaningful and broadly accepted terms, as well as ensuring that the relationships between the included terms be both accurate and accepted by the pertinent scientific community. For an ontology such as GO which is frequently used for functional enrichment analysis of genomic datasets, it is also important that terms be associated with specific gene products (coding and non-coding RNAs and proteins) in an empirically supported manner.

The Gene Ontology (GO) Consortium (GOC; http://www.geneontology.org) is a community-based bioinformatics effort. This Consortium develops, maintains and extends two interconnected resources: the Gene Ontology itself, and a database of GO annotations that associate specific gene products with concepts in the Gene Ontology. As of March 2016, there are >42,000 GO terms for describing concepts relevant to gene product function in a species-independent manner, providing not only comprehensive coverage of biological concepts but also community-wide agreement on how those should be used to describe gene functions across all organisms. The GO is organized into three aspects [38]: these are graph structures comprised of classes for molecular functions, the biological processes these contribute to, the cellular locations where these occur (cellular components), and the relationships connecting these classes. A ‘GO annotation’ describes the association between a gene product and an ontology class, as well as references to the evidence supporting the association. In most cases, a GO annotation is a statement about gene function, but because the GO cellular component aspect describes a “cellular and subcellular anatomy” it can have broader applications beyond the originally specified GO annotation usage of “where a gene product is active.” Thus an association of an exRNA to a cellular component term does not necessarily imply a function per se, though a functional role may of course later be discovered.

The GOC continuously provides enhancements to the ontology and annotation database, as well as its tools and policies, ensuring that the ontology and annotations are consistent, and accurately reflect the current state of biological knowledge.

In recent years, the GO has expanded the number and type of relationships used to connect terms within the ontology as well as between GO and other ontologies. A version of GO containing all relationships, including information from the Uber anatomy ontology (UBERON) [39], the Chemical Entities of Biological Interest ontology (ChEBI) [40], the Plant Ontology for plant structure/stage (PO) [41], the Phenotypic Quality Ontology (PATO) [42], and the Sequence Ontology (SO) [43], is called go-plus and is available at http://geneontology.org/page/download-ontology. The GOC also makes other versions of the ontology available at this site.

## Methods

As mentioned above, when we initiated our metadata efforts, there was a significant gap between the needs of the exRNA community and the terms and relationships present in GO. At that time, the only relevant terms in the Cellular Component branch were: ‘extracellular vesicular exosome’ (GO:0070062) and ‘prominosome’ (GO:0071914), neither of which was widely used in the exRNA community. In addition to lacking the most commonly used terms for extracellular vesicles, this version of GO lacked terms specific for exRNA-containing ‘Lipoprotein Particles’ (LPPs) and ‘Ribonucleoprotein Particles’ (RNPs).

### Defining exRNA-related terms based on community consensus

**Fig. 1:**
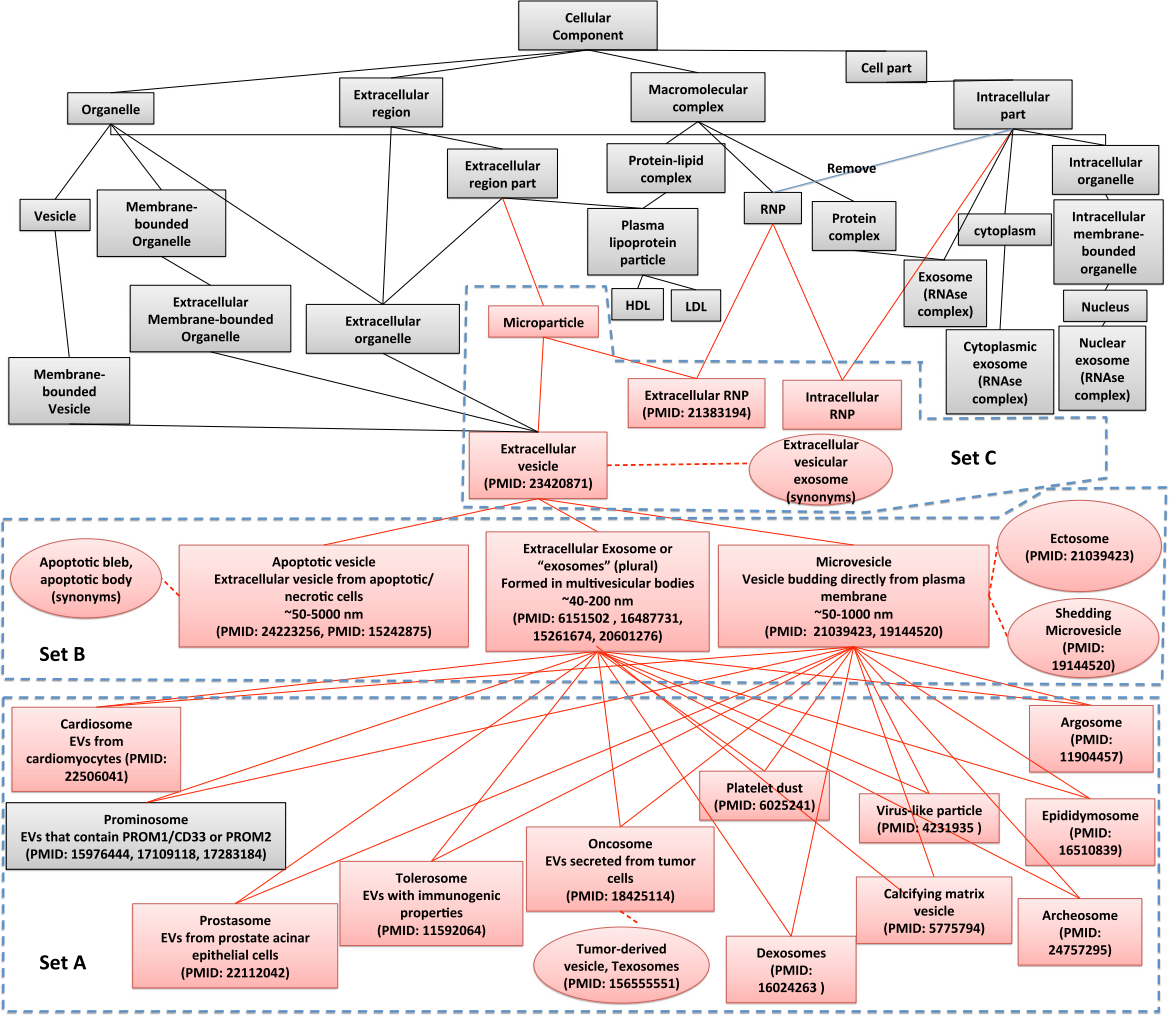
The three different options of extending GO to include ExRNA terms and relationships

Our goal was to extend the cellular component branch of GO to incorporate relevant and empirically supported terms and relationships that would be useful to and broadly accepted in the exRNA field, while both maintaining the internal consistency of GO and leveraging the knowledge previously encoded in the GO. Initially focusing on the major area of controversy (vesicles), we surveyed the literature for relevant references and sought input from domain experts, including the leadership of the International Society for Extracellular Vesicles (ISEV), the American Society for Exosomes and Microvesicles (ASEMV), and the ERCC. This process resulted in three alternative proposals, which ranged from inclusion of only the most general of terms, to inclusion of both general and moderately specific terms, to additional inclusion of highly specific terms (Fig. 1). As shown in Fig. 1, boxes represent GO terms, ovals represent synonyms, and lines connecting boxes represent the ‘is-a’ relationship (a dashed line connecting a box and an oval corresponds to a synonymous relationship). The gray boxes and black lines represent existing terms and relationships, while the red boxes and red lines represent the proposed terms and relationships. The option-3 set of terms/relationships is a subset of the option-2 set which is a subset of option 1. We then polled the membership of these societies either during their annual (ASEMV) or semi-annual (ERC Consortium) meetings or through an electronic survey (ISEV). The results of the ISEV poll are shown (Table 1), and are concordant with the consensuses reached by the other two groups: Option 2 (set B + set C) is the preferred choice.

**Table 1:** ISEV poll results

Rank	1	2	3	4
Option 1	16	86	89	21
Option 2	162	36	8	6
Option 3	33	83	91	5
Option 4	1	7	24	180

580 ISEV members received the poll, 212 answered it. The first question was to rank the four options by order of preference. As shown in Fig. 1, the proposed options were: Option 1 = Set A + B + C; Option 2 = Set B + C; Option 3 = Set C; Option 4 = none of the above. Number of answers for each

We then worked with the GOC team to incorporate the consensus recommendations, as well as terms relevant to LPPs, as supported by the literature. As part of the GO update, the GOC editorial team routinely adds new ontology classes, or modifies existing ones, based on requests from GO curators or from community experts. In the latter case, like our case here, changes to the ontology are proposed using a batch request upon direct consultation (see [44]).

### Adding gene product annotations

The GOC accepts contributions of annotations from external groups, and provides guidance in the ‘Documentation’ section of its website (http://geneontology.org). The ‘Documentation’ section includes details of the recommended file format, called GAF (Gene Association Format). Annotations submitted to the GOC undergo automated checks, followed by manual review by GO curators, in this case from the Protein Function Content team at EMBL-EBI.

As sources of gene product annotations in the exRNA context, ExoCarta [45] and Vesiclepedia [46] are online databases that catalogue proteins, RNAs and lipids identified specifically in exosomes and other extracellular vesicle types, respectively. Recently, ExoCarta was updated with ISEV minimal experimental requirements feature for definition of extracellular vesicles. In collaboration with the GOC editorial team, we have mapped the ExoCarta and Vesiclepedia gene products (coding and non-coding RNAs and proteins) onto their corresponding RNAcentral (http://www.rnacentral.org) and UniProt (http://www.uniprot.org/) identifiers using the newly extended GO Cellular Component terms.

### Incorporating new GO terms into exRNA Atlas as a use case

The exRNA Atlas is a central data repository developed and maintained by the DMRR that distributes data provided by the ERCC. The first public release of the exRNA Atlas, in early 2016, contained exRNA-seq profiles of over 500 samples generated by ERCC members analyzed uniformly using standard in-house analysis pipelines and quality-controlled using standards agreed by the Consortium. The exRNA Atlas browser enables efficient searching and sub-selection of exRNA profiles for retrieval and integrative analysis. To facilitate integration of the datasets into the exRNA Atlas, data and metadata standards have been developed by the Metadata Working Group of the ERCC. The metadata standards build on metadata data models we previously developed during the course of the NIH Epigenome Roadmap project, International Human Epigenome Consortium (IHEC) project, and in collaboration with the ENCODE3 project, and NCBI. The metadata include core objects such as biosamples, donors, experimental protocols, studies and analysis methods. The GenboreeKB document modeling system was used to define syntactic and semantic models for ontology-rich multi-faceted metadata based on the lightweight JSON syntax.

The latest release of the exRNA Metadata Standard associates various biomedical ontologies to both the attribute (key) values as well as with the attributes themselves and supports dynamic validation against ontologies available in NCBO Bioportal. More than twenty data elements within the metadata model are associated with ontologies, such as biofluids, disease types, anatomical locations, cell culture sources, cell lines, tissues, and other properties needed to describe biosamples and donors. A number of these elements reflect the clinical focus of the ERCC, defined by ontologies such as the Systematized Nomenclature of Medicine - Clinical Terms (SNOMEDCT) (https://www.nlm.nih.gov/research/umls/Snomed/snomed_main.html) for biofluid type as well as anatomical location of the biosamples and Human Disease Ontology (DOID) [47] for disease types. To effectively identify the source from which the exRNA is extracted, the new GO terms have been used to annotate the biosamples deposited in the exRNA Atlas.

## Results and discussions

### ExRNA terms and relationships added to GO

**Fig. 2:**
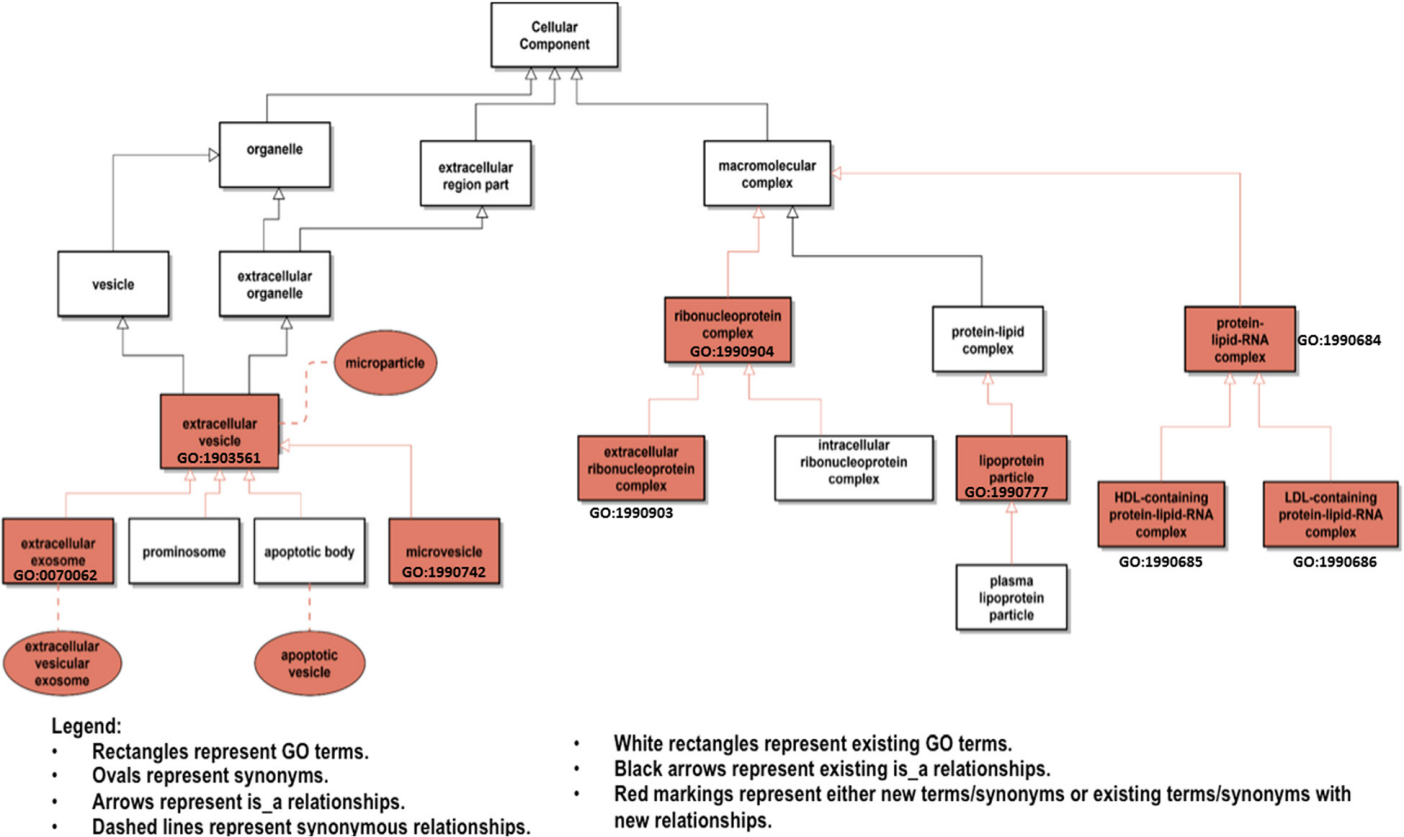
The list of exRNA-related terms, synonyms and relationships that have been added to GO

Based on the option-2 proposal shown in Fig. 1, the ERCC group worked with EMBL-EBI members of the GOC editorial team to add the proposed terms (including synonyms) and relationships to GO. Figure 2 shows these terms (with GO IDs) and relationships in red. Any future terms added to GO and representing types of vesicles that are part of the extracellular region will be automatically classified under GO:1903561 ‘extracellular vesicle’.

### Gene product annotations extracted from ExoCarta and Vesiclepedia

At the time of writing this manuscript, 18,695 coding (mRNA and protein) and 963 non-coding (ncRNAs) gene products (including isoforms) were annotated with the GO terms: ‘extracellular vesicle’, ‘extracellular exosome’, ‘apoptotic body’ and ‘microvesicle’ identified in different species (Table 2) from ExoCarta and Vesiclepedia. The Protein Function Content team at EMBL-EBI are currently working on importing annotations from ExoCarta and Vesiclepedia into the GOC. We believe that the gene product annotations extracted from these resources would further aid the scientific community in downstream cellular component enrichment analysis leading to understanding the molecular mechanism of extracellular vesicle biogenesis, as well as sorting and secreting under normal and pathophysiological microenvironments. While granular/specific annotations would be more informative, ontological inferencing can be applied to a given term (extracellular vesicle) to automatically retrieve its annotations and those associated with its descendant terms (e.g. extracellular exosome). GO query engines like QuickGO (http://www.ebi.ac.uk/QuickGO/) support this type of query. We anticipate that updating the gene products associated with each GO term will be an ongoing process.

**Table 2:** Summary of the gene product annotations extracted from ExoCarta and Vesiclepedia

GO-Term	GO-ID	Coding gene products (mRNAs or proteins)	Non-coding gene products (ncRNAs)
Extracellular exosome	GO:0070062	10,827	953
Apoptotic body	GO:0097189	168	
Microvesicle	GO:1903561	14,668	148
Extracellular vesicle	GO:1990742	4029	
		Total no. of unique coding gene products	Total no. of unique non-coding gene products
		18,695	963

### The use of GO terms to annotate exRNA metadata and implement faceted search within the exRNA Atlas browser

**Fig. 3:**
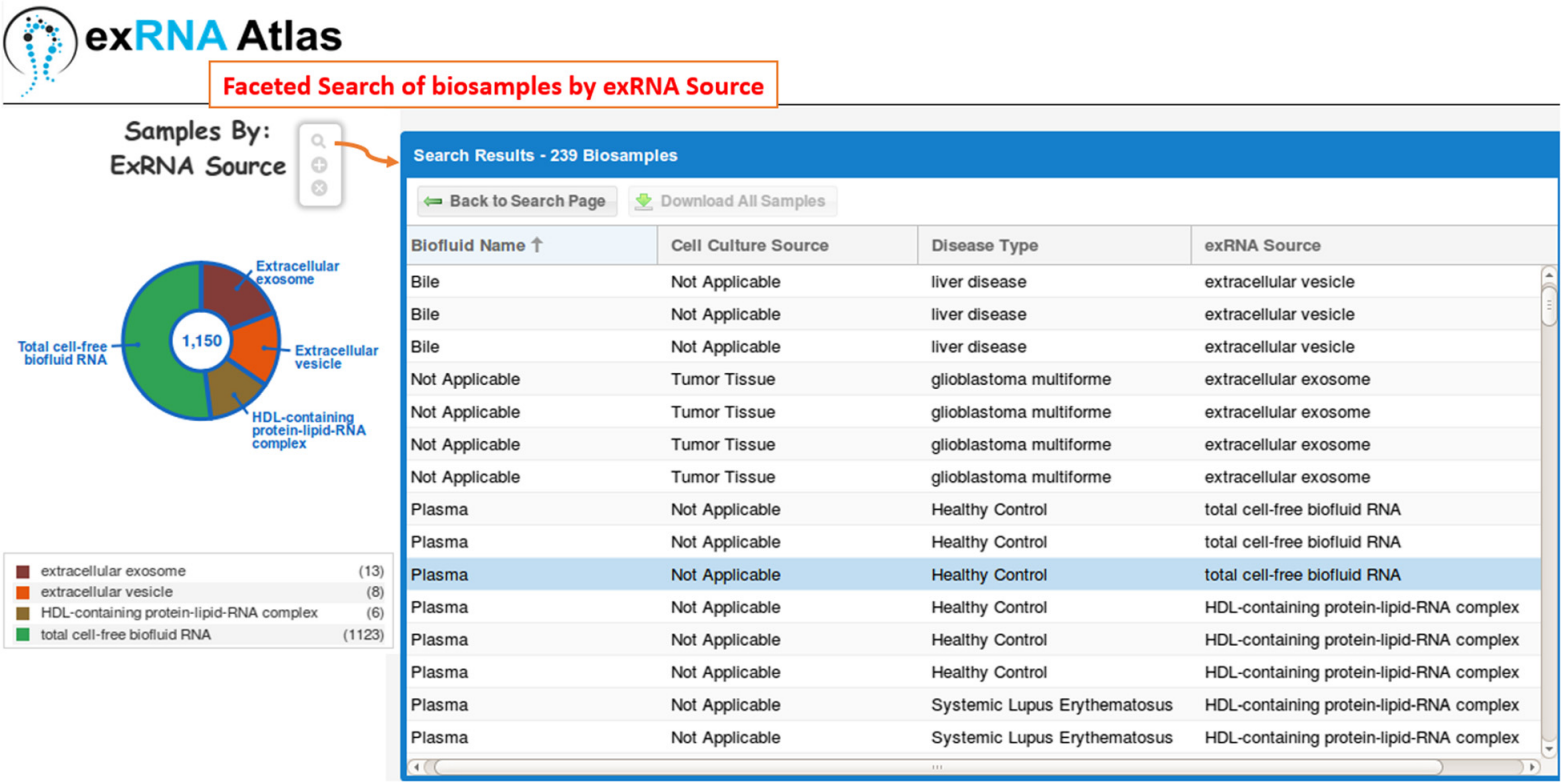
The new GO terms have been used to annotate the biosamples deposited in the ExRNA Atlas based on the *exRNA Source*. The figure is a screenshot of the faceted search of biosamples in the ExRNA Atlas using the *exRNA Source* metadata property (facet)

We have implemented a search interface that allows users to browse exRNA Atlas biosamples based on different facets of metadata, including the type of biofluid, cell culture source, disease, and exRNA source. The facets (categories of ontology concepts) provide an effective means for navigating/filtering a large set of exRNA profiles data based on different aspects of the samples. The metadata terms linked to ontologies in BioPortal have been augmented to include the newly defined exRNA terms (Fig. 3). By annotating the exRNA sources using these community-agreed terms and by exposing a new facet (exRNA source) through the ExRNA browser, we have enabled more precise biologically meaningful search of the ExRNA Atlas.

### Significance of our results and other possible extensions

These substantial changes to GO reflect the current state of the exRNA field, representing the current literature from the perspective of the consensus among three major professional societies in this field (ISEV, ASEMV, and ERCC). We believe that the new exRNA-relevant terms, together with their relationships to each other and with the existing GO terms, as well as the associated gene products, will be useful for annotating the increasing number of manuscripts and datasets being generated by the exRNA community. We also expect that they will prove useful for literature mining, data repository integration, and for computational analysis of exRNA data. Given the rapid developments in this field, we view the current version of the relevant areas of the ontology to be a work in progress. We anticipate frequent future amendments to incorporate as well as refine additional terms, relationships, and associated gene products as the community develops a more precise and comprehensive understanding of the biogenesis, biophysical properties, molecular composition, and functions of different types of exRNA-associated EVs, LPPs, and RNPs. To this end, the ERCC group aims to continually interrogate the literature and to solicit input from professional societies and the wider exRNA community. The ERCC has launched the ExRNA Portal (exrna.org), a publicly accessible website and community forum for dissemination of information, sharing of tools and data (primarily through the ExRNA Atlas), and aggregation of input. Future proposed amendments will be posted on the ExRNA Portal and will be subject to a public comment period. As described above, in the metadata associated with datasets submitted to the ExRNA Atlas, we permit and request depositors to not only tag datasets with the appropriate current GO terms, but to also include more precise keywords, so that over time, frequently used keywords can be evaluated as possible new GO terms.

In addition, our ExRNA Atlas use case (faceted search) shows the power of exploiting multiple ontologies in a sample annotation. For example, our metadata includes a description of extracellular RNAs isolated from different types of biofluids. Ontologies such as Uberon [48], NanoParticle Ontology [49], SNOMEDCT and Experimental Factor Ontology [50] contain concepts corresponding to different types of “Biofluid” (or “Bodily Fluid”). These concepts can be used alongside with the “extracellular space” concept in GO to describe extracellular RNAs that are isolated from different types of biofluids.

## Conclusions

In this manuscript, we have described an inter-community effort to make a substantial update to GO, focusing on terms and relationships pertinent to the new field of exRNA biology. We have demonstrated how these new GO terms can be used to annotate and search metadata associated with high-throughput datasets, such as RNAseq data. In addition, we anticipate that these terms will be useful as keywords for annotation and querying of the literature. We have also initiated a systematic, literature-supported process for associating gene products with each exRNA-related term, such that they can be used for cellular component enrichment analysis of omics datasets (e.g. RNAseq- and gene expression data). We recognize that fundamental questions regarding the composition, biogenesis, and functions of exRNA-containing EVs, lipoproteins, and ribonucleoprotein complexes remain to be answered. Therefore, we anticipate that frequent future updates to GO will be necessary to accurately reflect continued progress in this rapidly advancing field.

## Abbreviations

ASEMV: American Society for Exosomes and Microvesicles
ChEBI: chemical entities of biological interest
DMRR: data management and resource repository
ERCC: Extracellular RNA Communication Consortium
EV: extracellular vesicle
ExRNA: extracellular ribonucleic acid
GAF: Gene Association Format
GO: gene ontology
GOC: Gene Ontology Consortium
HDL: high density lipoprotein
ISEV: International Society for Extracellular Vesicles
LDL: low density lipoprotein
LPP: lipoprotein particle
mRNA: messenger ribonucleic acid
lncRNA: long non-coding ribonucleic acid
miRNA: micro ribonucleic acid
MVB: multivesicular body
NCBO: National Center for Biomedical Ontology
ncRNA: non-coding RNA
PATO: phenotypic quality ontology
PR: protein ontology
piRNA: Piwi-interacting ribonucleic acid
RNP: ribonucleoprotein
rRNA: ribosomal ribonucleic acid
SO: sequence ontology
tRNA: transfer ribonucleic acid
VHDL: very high density lipoprotein
VLDL: very low density lipoprotein
